# Murine Coronavirus Cell Type Dependent Interaction with the Type I Interferon Response

**DOI:** 10.3390/v1030689

**Published:** 2009-11-04

**Authors:** Kristine M. Rose, Susan R. Weiss

**Affiliations:** Department of Microbiology, University of Pennsylvania, School of Medicine, 36th Street and Hamilton Walk, Philadelphia, PA 19104-60761, USA; E-Mail: rosekris@mail.med.upenn.edu

**Keywords:** murine coronavirus, CNS infection, virus induced IFN induction, IFN signaling

## Abstract

Coronaviruses infect many species of animal including humans, causing acute and chronic diseases of many organ systems. Murine coronavirus, mouse hepatitis virus (MHV) infection of the mouse, provides animal models for the study of central nervous system disease, including encephalitis and demyelinating diseases such as Multiple Sclerosis and for hepatitis. While there are many studies of the adaptive immune response to MHV, there has until recently been scant information on the type I interferon (IFN) response to MHV. The relationship between MHV and the IFN-α/β response is paradoxical. While the type I IFN response is a crucial aspect of host defense against MHV in its natural host, there is little if any induction of IFN following infection of mouse fibroblast cell lines *in vitro*. Furthermore, MHV is relatively resistant to the antiviral effects of IFN-α/β in mouse fibroblast cell lines and in human 293T cells. MHV can, under some circumstances, compromise the antiviral effects of IFN signaling. The nucleocapsid protein as well as the nsp1 and nsp3 proteins of MHV has been reported to have IFN antagonist activity. However, in primary cell types such as plasmacytoid dendritic cells (pDC) and macrophages, IFN is induced by MHV infection and an antiviral state is established. Other primary cell types such as neurons, astrocytes and hepatocytes fail to produce IFN following infection and, *in vivo*, likely depend on IFN produced by pDCs and macrophages for protection from MHV. Thus MHV induction of IFN-α/β and the ability to induce an antiviral state in response to interferon is extremely cell type dependent. IFN induced protection from MHV pathogenesis likely requires the orchestrated activities of several cell types, however, the cell types involved in limiting MHV replication may be different in the liver and in the immune privileged CNS.

## 1. Introduction

Coronaviruses are a family of large positive-sense RNA viruses that cause a wide range of veterinary and human diseases. Coronaviruses are divided into three groups, with group I and II viruses infecting mammals and group III viruses infecting avian species [1]. Human coronaviruses (HCoV), HCoV-229E (group I) and HCoV-OC43 (group II), cause approximately 5–30% of all human respiratory infections [1,2]. In late 2002, Severe Acute Respiratory Syndrome associated coronavirus (SARS-CoV) infected more than 8,000 people resulting in approximately 750 deaths [3–5], demonstrating that human coronaviruses can also cause more serious disease in humans. Although SARS-CoV has not re-emerged in humans since the initial outbreak, other than a few remote incidents, isolation of SARS-related viruses in civets and several other animals [6–8] and more recently in bats [9,10] suggests that an animal reservoir of the virus exists. The identification of two new human coronaviruses since the SARS epidemic, the group I HCoV-NL63 [11,12] and the group II HCoV-HKU1 [2,13], associated with respiratory illnesses have also added to the need to further our understanding of coronavirus pathogenesis.

Mouse hepatitis virus (MHV), the prototype group II coronavirus, has long been used as a model for the study of coronavirus replication and pathogenesis. MHV strains exhibit various organ tropisms as well as pathogenic potentials. MHV strains can be divided into two groups according to patterns of tropism. One group of strains (MHV-D, -Y, -RI, -S/CDC, LIVIM, and DVIM) is enterotropic; infections of mouse colonies with these strains generally produce infections confined to the GI tract [14]. The other group (MHV-1, -2, -3, -4 (or JHM) and -A59)) contains polytropic strains; experimental infections of rodents with these strains provide animal models for humans diseases such as hepatitis, encephalitis, demyelinating diseases such as multiple sclerosis (MS) and most recently respiratory disease such as SARS [15]. The strains used most commonly for pathogenesis studies are the neurotropic JHM, the hepatotropic/neurotropic A59 and hepatotropic MHV-3 [1].

**Figure 1.**
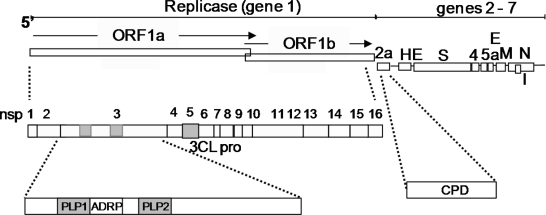
Schematic of MHV genome.

MHV has a 32kb single stranded positive sense RNA genome [16]. The structural proteins, spike (S), matrix (M), and envelope (E) are all found within the viral envelope, which surrounds the nucleocapsid, containing genome RNA in association with the nucleocapsid protein (N). There is an additional structural, yet non-essential protein, the internal protein (I), which is of unknown function [17]. Some MHV strains, including some JHM isolates express the hemagglutinin-esterase protein (HE) which forms smaller spikes on virions [18] and enhances neurovirulence, when paired with specific viral genes [19]. During infection, in addition to the structural genes, there is expression of the 16 replicase proteins (encoded in the 5′ two thirds of the genome), as well as several nonstructural proteins (encoded in ORFs 2a, 4, and 5a), which currently have unknown functions (Figure 1) [1].

While there is much known about the adaptive immune response to MHV [1,20–22], until recently, there had been limited information about the IFN-α/β response induced by MHV infection either *in vivo* or *in vitro* [23–26]. However, in the last few years there has been an evolving literature on this subject, which will be reviewed here. The two main target organs for the most commonly studied murine coronavirus strains are the central nervous system (CNS) and the liver. An interesting aspect to MHV infection is that host immune responses in these two organs may be quite different due to the immune privileged status of the CNS and the unique toleragenic environment of the liver [27]. There have been several recent reviews of the innate immune response to coronaviruses, focusing on SARS; this review will focus mainly on MHV [28–31].

## 2. Results and Discussion

### Virus induced innate immune response

The use of reverse genetics to construct recombinant viruses has enabled our lab and others to investigate the interactions between virus and host and identify important virulence factors leading to CNS and liver disease [1]. Understanding the outcome of the initial host-pathogen interaction requires identification of viral molecular patterns that are perceived as foreign by pattern recognition receptors (PRRs) and characterization of the ensuing inflammatory response. The immediate responses to infection include synthesis of cytokines, chemokines, antiviral proteins, and activation and recruitment of innate immune cells (DCs, macrophages, neutrophils, NK cells), which work together to limit virus replication and initiate the virus-specific adaptive response. The importance of the type I interferon (IFN) response consisting of the cytokines IFN-α/β, in control of virus replication *in viv*o, was demonstrated by the observation of enhanced viral replication, dissemination, and increased mortality in IFN-α/β receptor knockout mice (IFNAR-/-) infected with a wide variety of viruses with variable tropisms and disease outcomes [32].

The cellular innate immune response recognizes pathogen-associated molecular patterns (PAMPS) expressed by viruses such as dsRNA. Coronaviruses replicate in the cytoplasm where they produce large amounts of dsRNA [33,34] which can potentially be recognized by PRRs. Toll-like receptors (TLR), for example TLR-3 and TLR-7 which sense RNA, are localized to the endosomal membrane and recognize virus entering through endocytosis. In addition, RIG-I like helicases (RLH), RIG-I and MDA5, are located in the cytoplasm and recognize virus entering via the cell surface and replicating in the cytoplasm. MHV enters cells through both receptor-mediated fusion with the plasma membrane and by endocytosis [35,36] facilitating recognition by either TLRs or RLHs. There is considerable evidence that different PRRs recognize discrete RNA structures, which allows discrimination of viral RNA species produced only from a specific family of viruses [37,38]. In addition, viruses may be recognized by distinct PRRs in different cell types. Upon recognition of dsRNA or ssRNA substrates, RIG-I and MDA5 interact with IPS-1 (also called MAVS, Cardif, and VISA) on the mitochondrial membrane, which initiates a signaling cascade through the kinases TBK1 and IKKε. These kinases phosphorylate and activate the cytoplasmic transcription factor IRF-3 that translocates to the nucleus and localizes on the IFN-β promoter/enhancer along with activated NFκB and other transcription factors to initiate IFN-β transcription. Alternatively, engagement of TLRs leads to recruitment of TIR domain containing adaptors TRIF and MyD88 to initiate signal transduction from TLR-3 and TLR-7, respectively. A distinct series of ubiquitin ligases and kinases lead to the activation of IRF-3, NFκB, and AP-1 and their transport to the nucleus to assemble and initiate IFN-β transcription (reviewed in [39]).

Secreted IFN-α and -β bind to a distinct IFNAR receptor (IFNAR1/IFNAR2) expressed on the surface of most nucleated cells. Ligand binding induces receptor oligomerization followed by phosphorylation of STAT1 and STAT2 by IFNAR associated kinases, JAK1 and TYK2, respectively. STAT1 and STAT2 dimerize and associate with the transcription factor IRF9 to form the ISGF3 heterotrimer. Formation of this complex drives ISGF3 nuclear transport and binding to the IFN-stimulated response element (ISRE), found in promoters of most IFN-stimulated genes (ISGs), which enhances transcription (reviewed in [40]).

During the initial infection, a competition between the virus’ ability to antagonize IFN production/signaling and the cell-type specific IFN response largely determines disease pathogenesis and viral tropism. Until viruses synthesize enough IFN antagonists to limit the IFN response, they must alter the mode or kinetics of replication to limit production of potential viral inducers of IFN (dsRNA and ssRNA). Consequently, any slight defect in entry, RNA synthesis or viral packaging could drive the balance in favor of host control, which enables establishment of an adaptive response leading to viral clearance. Thus, viruses often employ multiple strategies to evade the antiviral interferon system including: (i) inhibition of global protein synthesis; (ii) prevention of IFN production by limiting production or recognition of PAMPs; (iii) interfering with PRR expression or activity; (iv) blocking IFN signaling; (v) inhibition of interferon stimulated genes (ISG) antiviral activity or (vi) a replication strategy that is resistant to the antiviral effects of IFN.

### Innate immune response to virus infection in the CNS

The adaptive immune response in the immune privileged CNS is generally considered to be different from that in the peripheral organs, leading to the speculation that the type I IFN response may be different as well [32]. This is supported by the observation that the major type I IFN producer cell type plasmacytoid dendritic cells (pDC), are not present in the brain parenchyma and IRF-7, the transcription factor mediating IFN-α expression in pDCs is not constitutively expressed at a high level in the CNS [41]. Despite this finding an important role for type I IFN was inferred from the increased neurovirulence of numerous viruses following infection of mice genetically deficient in the expression of IFN receptor (IFNAR-/-) [32]. Thus, studies have been carried out to determine the role of the type I IFN response in viral infections of the CNS, the cell types responsible for the expression of IFN in the CNS and the response of individual cell types to IFN. Recent reports demonstrate that most major cell types can express IFN following infection with a selection of viruses, but not all viruses examined. Delhaye *et al*. [41] showed that infection of mice with either Theiler’s virus (a picornavirus) or La Crosse (bunyavirus), induced IRF-7 expression in the brain. Using immunohistochemistry to localize IFN-α/β producing cells, following infection, several parenchymal CNS cell types, including macrophages, ependymal cells and a small percentage of neurons, were shown to express IFN-α/β; some, but not all of these cells were also expressing viral antigen. Furthermore all of the CNS cell types analyzed, including neurons, astrocytes, oligodendrocytes, macrophages, ventricular epithelial cells, vascular endothelial cells, responded to IFN as evidenced by the induction in the CNS of at least some interferon stimulated genes (ISGs), including IRF-7 and Mx protein.

### Overview of the interaction of MHV and IFN-α/β pathway

Studies of MHV in cultured cell lines generally demonstrate that MHV either does not induce type I IFN [33,42] or induces IFN protein [23] or IFN mRNA only [43] to a very limited extent and only very late after infection. This is in contrast to other viruses, for example Sendai (SeV) or Newcastle disease virus (NDV), which can induce significant amounts of IFN in these same cell lines. Despite this lack of induction, MHV was unable to block IFN induction by other viruses or by poly I:C, leading to speculation that MHV replication cycle somehow involved sequestering of RNA from the pattern recognition receptors (PRR) [33,42] rather than actively compromising the response. As will be described below, induction of IFN IFN-α/β is cell type specific and MHV induces IFN-α/β in only a minority of cell types that have been examined.

In fibroblast cell culture, in addition to failing to induce significant type I IFN, MHV is relatively resistant to pre treatment of mouse fibroblast cell lines with IFN-α/β [43,44] suggesting that it is resistant to the antiviral effects of IFN treatment. MHV may act by either inhibiting signaling or blocking specific functions of interferon stimulated gene products in fibroblast cultures. However, IFN is crucial during infection *in vivo* in limiting virus replication and spread [45–48]. This underscores the cell type specific differences in the interaction of MHV with the type I IFN response.

We will review the literature on the interactions of MHV with the type I IFN system both in infections *in vivo*, in the CNS and the liver, and *in vitro* with an emphasis on the cell type specific aspects of the interactions. This will be followed by a discussion of MHV proteins that may play a role in immune evasions strategies.

### Sensitivity of MHV to antiviral effects of IFN-α/β is cell-type dependent

Early studies by Virelizier *et al*. [49] revealed that IFN was important for protection from MHV-3 induced fulminant hepatitis. Injection of mice with anti-interferon antibodies to decrease endogenous interferon levels caused acceleration of MHV-3 induced hepatitis and death in susceptible mice and acute lethal disease in resistant mice. In support of this observation, mice treated therapeutically with recombinant IFN-α or –β alone or in combination with ribavirin, a therapy used to limit hepatitis C virus replication in the liver, increased survival following infection with MHV-2 (a highly hepatotropic strain) [50–52], and decreased viral titers [23]. In addition, IFN-α2 selectively expressed in the liver using a helper-dependent adenoviral delivery system, protected against acute hepatitis in MHV-3 challenged C57BL/6 mice [53]. Collectively, these data suggest that exogenous IFN-α/β restricts MHV replication in the liver and protects against MHV-induced hepatitis. Benefits of IFN-α/β treatment, however, have not been investigated in the context of MHV infection of the CNS.

**Figure 2.**
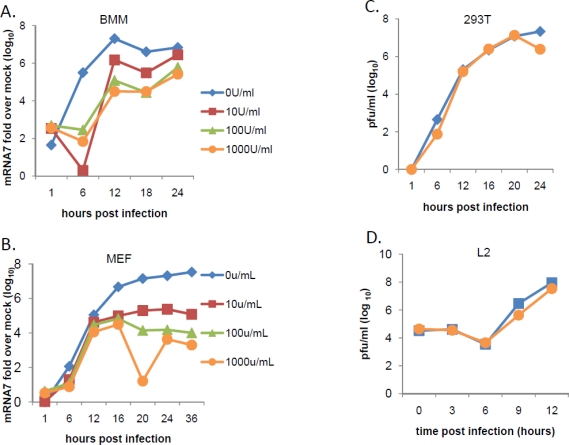
A59 replication is inhibited in response to IFN-β in BMM and MEF. Primary cells or cell lines were treated with indicated concentrations of IFN-β for 16 hours prior to infection with MHV-A59 at MOI=10. A59 was incubated with cells at 370 °C for 1h hour after which virus was removed and cells washed three times in excess of PBS before adding fresh media and returning cells to 37 °C incubation. (A,B) Total RNA from infected cells was isolated at several time intervals post infection and analyzed by qRT-PCR for viral mRNA7. Values are normalized to β-actin and expressed as fold over mock infected cells. (C,D) Supernatants were collected from infected 293T cells transiently expressing MHV-R (CEACAM1a) or L2 cells at times indicated and viral titers were determined by plaque assay in L2 cells. Data are representative of at least two independent experiments.

The relationship between MHV and the role of IFN-α/β response is paradoxical. Early reports [23] and the more recent studies of *in vivo* infections mentioned above [45–48] demonstrate a critical role of IFN-α/β response in protection from MHV infection. However, replication of MHV strains A59, JHM and MHV-2 was only marginally impaired in L2 and 17Cl-1 cells [43,44] and 293T (Figure 2) treated 16h prior to infection with high doses of IFN-β, while replication of Newcastle Disease virus (NDV) was completely inhibited. This indicates that L2 cells can mount a functional IFN response; however, MHV evades the antiviral activities by an unknown mechanism. Despite resistance to IFN, MHV is unable to protect NDV from the antiviral effects of IFN when the two viruses are co-infected in L2 cultures [43]. Establishing MHV infection 3 hours prior to IFN treatment; however, releases a block that allows SeV to replicate in the presence of IFN-α or –β. Under these conditions MHV appears to provide protection from IFN by inhibiting virus- and IFN-induced expression of a subset of ISGs (K. Rose *et al*., manuscript in preparation). In contrast, a substantial reduction in MHV replication occurs in IFN-α or -β treated MEFs (Figure 2), primary bone marrow-derived macrophages (BMM) and conventional DCs (cDCs) [47,54,55]. These data provide evidence that the sensitivity of MHV to IFN is cell-type dependent.

As suggested by Zurney *et al*. [56], cell-type differences in initial responses to virus infection may depend to some degree on basal expression levels of IFN signaling molecules and transcriptions factors involved in the IFN response. This group proposed that cardiac myocytes, which are not readily renewed, express high endogenous levels of IFN-β, leading to higher expression of ISGF3 complexes and ISGs. In this steady state, cardiac myocytes are already carrying weapons to limit virus replication. Similarly, neurons are a nonrenewable cell type in the CNS, which has led to speculation that neurons would require a similar strategy to limit viral-mediated cell lysis or innate immune cell cytotoxicity [57].

### Role of type I IFN signaling during MHV infection in the CNS and liver *in vivo*

It is clear from many studies that CD8T cells, with the help of CD4T cells, are critical for clearance of MHV from the CNS and the liver. Supporting the notion that a CD8T cell response is crucial to protection from MHV in the CNS, the most neurovirulent variant of JHM (MHV-4) fails to induce an effective T cell response in the CNS [58–60]. The T cell response to MHV peaks at about five days post infection. During the first few days post infection there is a robust innate response including macrophage migration and secretion of chemokines, the quantity and quality of which is MHV strain dependent [20,59–61]. In order to investigate a role for type I IFN in defense against MHV before an effective T cell response develops, infections were carried out in the absence of IFN signaling, that is, in IFNAR-/- mice [45,46,48]. These studies used MHV strains A59 (neurotropic and hepatotropic) or JHM (neurotropic only) and infection by either intracranial (IC) or intraperitoneal (IP) routes of infection. Infection of IFNAR-/- mice, with low doses of either strain by either route of inoculation were highly lethal and led to increased levels of infectious virus in the usual target organs as well as spread to other organs not usually affected and dramatically accelerated clinical signs and mortality.

Ireland *et al*. [45] carried out IC infections of six-week-old C57Bl/6 and congenic INFAR-/- mice with the V2.2-1 attenuated glial tropic variant of JHM. Infected IFNAR-/- mice experienced increased mortality and virus was able to spread more extensively among glial cell types and to neurons, not usually infected with this strain. Furthermore infection in the absence of IFN signaling resulted in more rapid mortality and was able to convert infection with a nonpathogenic strain to a lethal infection. The observation that in the absence of IFN signaling, CD8T cells were induced, were functional as evidenced by the ability to secrete IFN-γ and were recruited to the CNS underscores the importance of the type I IFN response even in the presence of a robust T cell response. Accompanying increased pathogenesis, there were other alterations in the immune response including increased CD45+ cells in the CNS, enhanced neutrophil response and some changes in cytokine and chemokines in the CNS as well as reduced MHC class I expression on microglia.

Using bone marrow chimeras and transgenic mice with cell type specific abrogation of IFNAR expression, Cervantes-Barragan *et al*. [47] showed that type I IFN signaling was most important on LysM+ macrophages and to a lesser degree on CD11c+ DC to control viral replication and spread and to limit hepatitis regardless of route of inoculation (intraperitoneal (IP) or intranasal (IN)). Although to a lesser extent, IFNAR expression on parenchymal cells also contributes to limiting virus replication and lethal hepatitis when virus is introduced IP. In contrast, using the intranasal (IN) route of inoculation to compare A59 infection of wild type and mice with cell type specific abrogation of IFNAR, these authors demonstrated that loss of signaling on either LysM+ macrophages, CD11c+ DC, CD19+ B cells, or CD4+ T cells had no significant effect on replication in the brain despite the limitation of replication in peripheral organs. Since IFNAR signaling is essential for control of A59 spread of virus following IC inoculation as described above [45,48], these data imply that IFN signaling on parenchymal cells may have a greater impact on spread of virus within the brain, depending on the route of inoculation and the initial cell types infected. In support of this, a recent publication by Detje *et al*. [62] used targeted knockout of IFNAR from neuroectodermal cells to emphasize the necessity of IFNAR expression in the glomerular layer of the olfactory bulb to prevent intranasally introduced VSV from replicating and spreading in the CNS. These data highlight the importance of IFN signal transduction in specific cells within the CNS as a means to control initial virus infection and spread and remains an interesting avenue to be investigated in the context of MHV infection.

### MHV induction of IFN-α/β *in vitro*

**Figure 3.**
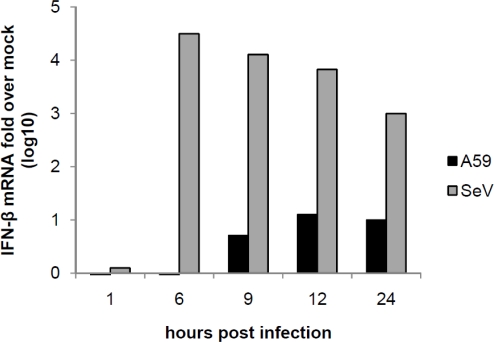
A59 induces low levels of IFN-β mRNA in mouse embryonic fibroblasts (MEFs). MEF were mock infected or infected at MOI=1 with A59 or SeV and RNA was isolated at indicated times. Total RNA was analyzed by qRT-PCR with primers specific to IFN-β mRNA. Values represent IFN-β mRNA levels relative to those in mock infected MEFs. Data are representative of four independent experiments.

Early observations that various strains of MHV either failed to stimulated IFN-β production or induced only small amounts of IFN-β at late times post infection (24 hours) in several rodent cell lines [24] led to investigations into possible innate immune evasion mechanism of this murine coronavirus. Consistent with these results, several labs confirmed that murine and human fibroblasts cells lines (17Cl-1, L2, 293T, and L929) and primary mouse embryonic fibroblasts (MEFs) (Figure 3) productively infected with MHV-A59 or –JHM displayed little to no IFN-α/β production [33,42,43]. Yet, Sendai virus (SeV) infection of these same cell lines induced IFN-β mRNA and protein as early as three hours post-infection [43]. Not surprisingly, in several MHV infected fibroblast cell lines, NFκB and IRF-3 were not localized to the nucleus up to 12 hours post-infection [33,42,43]. A small percentage of MHV infected cells had nuclear localized IRF-3 at 24 hours post infection which corresponded to small amounts of IFN-β mRNA (but not IFN-β protein) being produced [43]. Similarly, lack of IFN-α production in L-ACE2 cells [33] correlated with early IRF-3 translocation to the nucleus of SARS CoV infected cells followed by subsequent cytoplasmic localization due to an inability to interact with the transcription factor CBP which is needed for nuclear retention and initiation of IFN-β transcription [63].

Although the mechanism by which MHV evades recognition by cellular PRRs is unknown, MHV infection of fibroblast cells does not inhibit IFN-β induction by RIG-I or MDA5 agonists. SeV or poly I:C activation of a reporter plasmid containing the IFN-β promoter enhancer or production of IFN-α or -β mRNA following SeV infection is not diminished in cells infected with MHV [33,42,43]. Not surprisingly, activation of downstream signaling events, such as IRF-3 [33,42,43] and NFκB translocation [42], following SeV infection or poly I:C transfection are not altered by MHV infection. These observations suggest that MHV, unlike many other viruses including SARS CoV [30], does not inhibit RIG-I or MDA5 from recognizing RNA substrates or prevent the signaling events leading to IFN-β production in fibroblast cells. Taken together these observations suggest that the IFN-β production pathway is intact in fibroblast cell lines and that there is a selective block in the cellular machinery involved in MHV- stimulated IFN production. Similarly, a recent publication demonstrated that immortalized MEFs become permissive to myxoma virus infection due to a lack of induction of IFN-α/β in response to infection despite the production of IFN-α/β in response to dsRNA and dsDNA, indicating the presence of a fully competent TLR3, RLH, and dsDNA signaling pathways [64].

**Figure 4.**
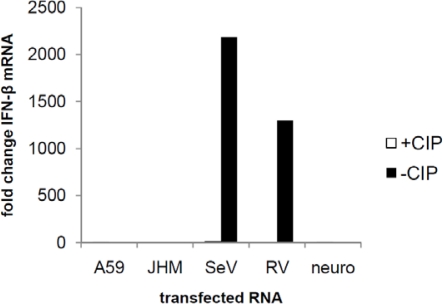
Transfection of 293T with MHV RNA does not induce IFN-β mRNA. RNA was isolated from 293T transiently expressing MHV receptor (CEACAM1a) 24 hours post infection with A59, JHM or SeV or neuroblastoma cells mock infected or infected with rabies virus (RV). In order to assay the ability of the isolated RNA to induce IFN-β mRNA, 293T cells were transfected using Fugene 6 reagent (Roche) with 1μg of total RNA that was treated with calf intestinal phosphatase (+CIP) or left untreated (−CIP). IFN-β mRNA levels in transfected 293T was analyzed using qRT-PCR 24h post transfection. mRNA values are expressed as fold change over 293T cells transfected with mock infected RNA. Data represent three independent experiments.

The inability to block IFN-α/β induced by other PAMPs and lack of IFN-α/β induction despite detection of large amounts of dsRNA generated by coronaviruses in infected cells [33,65] has led to speculation that the viral RNA is sequestered in a protected site that excludes detection machinery including PRRs. MHV, like many other RNA viruses, replicates on double membrane vesicles [66] that are hypothesized to be this privileged site [42]. Transfection of 293T cells with RNA from MHV infected cells which contain high levels of viral dsRNA does not induce IFN-β while transfection of RNA from Rabies virus (RV) infected neuroblastoma cell lines or SeV infected 293T cells induces significant amounts of IFN-β mRNA (Figure 4). IFN induction was dependent on 5′ triphosphate ends on viral RNA since treatment with calf intestinal phosphatase eliminated recognition of viral RNA (Figure 4). These results suggest that even when MHV RNA is introduced directly into the cytoplasm, RLH are still unable to sense MHV RNA. However, in interpreting these results, it must be kept in mind that alterations in the conformation or structure of the RNA that may occur during the isolation process may prevent it from binding to RLH.

**Figure 5.**
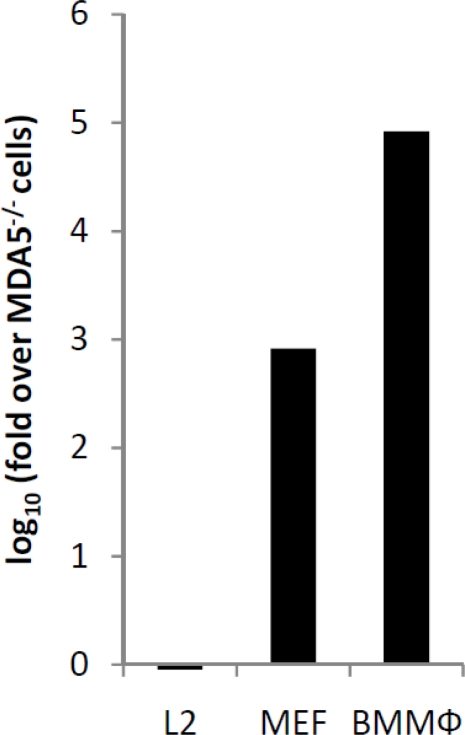
MDA5 mRNA expression is undetectable in fibroblasts that do not induce IFN-β in response to MHV infection. Total RNA isolated from L2, MEF, and WT (129x1/SvJ) and MDA5-/- BMM and analyzed by qRT-PCR for MDA5 expression. Values are normalized to β-actin and expressed as fold over MDA5-/- BMM. Similar results were obtained from three independent experiments.

Consistent with the above observations in fibroblast cultures, little to no detectable IFN-β mRNA and no protein was produced up to 24 hours post infection in primary cell cultures of neurons, astrocytes, and hepatocytes, all of which produce large amounts of virus both *in vitro* and *in vivo* [48]. However, *in vivo* neurons are capable of producing IFN-β in response to infection by the neurotropic viruses, Theiler’s and La Crosse [41], and primary neurons produce IFN-β in response infection by RNA viruses such as Sindbis [48] and West Nile Virus [67]. We propose that MHV may possess a mechanism to avoid detection by PRRs in certain cell types. Several possible scenarios may explain MHV evasion of detection: (1) MHV RNA is modified in certain cell types to prevent recognition by PRRs. Picornaviruses protect the 5′ of genomic RNA by covalently linked VPg protein and Borna disease virus expresses a phosphatase that converts the 5′ triphosphate, needed for RIG-I recognition, to monophosphate [68,69]. (2) Basal levels of PRRs may not be sufficient for detection of virus. In the cell types evaluated thus far, levels of MDA5 mRNA expression correlate with IFN-β production following MHV infection. Thus, MDA5 mRNA is barely detectable in L2 cells which correlates with the inability of L2 cells to induce IFN-β in response to MHV infection, while BMM that produce significant levels of IFN-β in response to MHV infection [48], express approximately 10^5^ fold higher levels of MDA5 mRNA (Figure 5). MEFs express intermediate levels of MDA5 mRNA, consistent with weak induction of IFN-b mRNA (Figure 3) (3) PRRs are attenuated in fibroblasts by regulators such as DAK, LGP2, ISG15, RNF125 [70] or may be destroyed by protease cleavage as observed during picornavirus infection [71]. (4) Inhibition of IFN production by MHV may not be complete, allowing for induction of INF by alternative pathways used by SeV or poly I: C.

Despite the lack of type I interferon response in fibroblast cultures as well as in several primary neural cell cultures, MHV infection does induce IFN-α/β in spleen-derived plasmacytoid DCs (pDCs) [46] and *ex vivo* microglia and macrophages isolated from the infected brain as well as in bone marrow derived macrophages *in vitro* [48]. Thus any explanation for the apparent lack of recognition of MHV RNA by PRRs must account for the observation that this varies with cell type and may have to do with factors such route of entry of virus, PRR utilized or localization within the cell of viral RNA in transcription complexes.

### Induction of IFN-α/β expression *in vivo* is cell-type specific

Several studies have investigated the induction of IFN-α/β following *in vivo* MHV infection with the neurotropic MHV strain, JHM or the hepatotropic/neurotropic strain, A59. Both A59 and JHM induced IFN–α/β mRNA in the brain following intracranial (IC) infection [45,48,59,72]. Rempel *et al*. [59], reported the highly neurotropic JHM induced more type I IFN mRNA, as measured by an RNAse protection assay, for a longer period of time post infection than the weakly neurotropic A59. However, Roth-Cross *et al*. [43], using qPCR to quantify mRNA and ELISA to quantify protein, were unable to detect differences in the induction of IFN-β following IC infection of the CNS with the weakly neurotropic A59 versus the highly neurovirulent JHM. In general, these authors observed IFN-β mRNA and protein in the brain and liver correlated with replication in that organ, thus JHM failed to induce IFN in the liver along with its failure to replicate in that organ [43].

In a study of MHV induced hepatitis, following infection with A59 by the intraperitoneal (IP) route, IFN-α was detected in the serum. Interestingly, plasmacytoid dendritic cells (pDCs) were identified as the major source of secreted IFN-α. In fact, significantly enhanced hepatitis and virus dissemination ensued in mice that were depleted of pDCs by the antibody, α-mPDC-1; emphasizing the importance of pDC derived IFN-α in host control of MHV when the virus is inoculated IP. Conversely, expression of IFN-α/β by *in vitro* infected cDCs was detected only very late after infection [46]. Following IC infection with A59, Roth-Cross *et al*. found macrophages and microglia recovered from the CNS of A59 infected wild type mice were expressing IFN-β, while *in vitro* cultures of primary neurons, astrocytes and hepatocytes, three cell types infected by MHV *in vivo,* failed to produce detectable amounts of IFN [48]. Together these data support the conclusion that MHV induction of IFN is highly cell type dependent. Furthermore, these studies demonstrate that DCs and macrophages play dual critical roles in secondary lymphoid organs in 1) protection from infection both by restriction of replication and also by secretion of IFN-α and -β for protection of other cell types [47].

**Figure 6.**
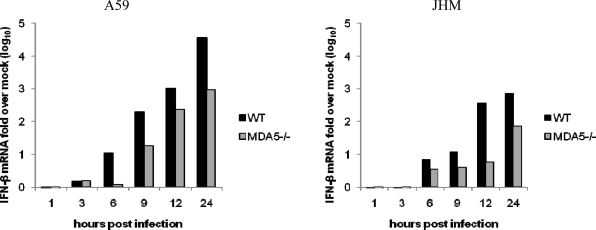
MDA5 expression is partially responsible for recognition of MHV in bone marrow-derived macrophages (BMM). WT or MDA5-/- BMM were isolated from 129x1/SvJ mice and infected with A59 (MOI=1) or JHM (MOI=0.1) and total RNA was isolated from infected cells at indicated times. IFN-β mRNA levels were determined by qRT-PCR using IFN-β specific primers and values are expressed relative to levels in mock infected BMM. Data are representative of two independent experiments.

Using bone marrow-derived macrophages (BMM) with specific knockouts in PRRs, MDA5 was found to play a significant role in the recognition of MHV-A59 and –JHM [48]. However, since appreciable amounts of IFN-α and –β mRNA are still produced in MDA5-/- BMM, we suspect that other PRRs can detect MHV generated PAMPs during the course of MHV infection of BMM (Figure 6). Further experiments are needed to identify all the PRRs involved in interferon induction in BMM. In pDCs TLR7 is essential for induction of IFN-α and –β in response to MHV [46].

### MHV encoded IFN antagonists

The observation that MHV both fails to induce IFN and is relatively resistant to the antiviral effects of IFN signaling in fibroblasts suggests the genome may encode antagonists of IFN synthesis and signaling. Indeed this has been the case for the human SARS related coronavirus [29,73]. As described below there are fewer examples of MHV proteins with direct effects on either IFN induction or signaling. It is not always easy to separate out direct viral antagonism of IFN from other activities that may effect viral pathogenesis and have indirect effects on the induction of IFN or the ability of the virus to replicate in the presence of IFN. Also, MHV proteins may function in concert to evade IFN responses, thus overexpression of individual proteins may not reveal IFN antagonist properties.

### Nucleocapsid

Ye *et al*. [44] reported anti-IFN activity was associated with the nucleocapsid (N) protein of MHV, strain A59, as evidenced by the functional replacement of the E3L protein of vaccinia virus, a known antagonist of IFN signaling. In addition to activation of IFN, the PKR and 2′5′ OAS are the main antiviral pathways activated directly by dsRNA. Expression of N was able to rescue vaccinia virus ΔE3L (VVΔE3L) from the antiviral effects of IFN and prevent degradation of host RNA suggesting N is able to antagonize the activity of RNase L. Consistent with this was the observation that MHV fails to induce the antiviral 2′5′ OAS/RNase L pathway. Similarly, MHV infection in the presence or absence of IFN does not activate PKR as evidence by lack of eIF2α phosphorylation and only minor inhibition of protein synthesis observed in 17Cl-1 mouse fibroblasts. This evidence suggests that MHV is also able to prevent activation of PKR. Yet, MHV N expressed in VVΔE3L failed to prevent activation of the PKR pathway in infected HeLa or 17Cl-1 cells. These IFN antagonistic properties of N do not appear to be conserved in all group II coronaviruses since over expression of the N protein of SARS, blocked induction of IFN-β but was unable to compromise IFN signaling events [74]. Thus the N proteins of MHV and SARS may interact differently with the host cell IFN-β response. Alternately N may behave differently when expressed from a heterologous viral genome or over expressed from a plasmid.

### Nsp1

The N terminal cleavage product of the polyprotein encoded in the replicase gene of MHV, nsp1 (p28), was shown to have a role in pathogenesis *in vivo*. A mutant of MHV-A59 with a 99 nucleotide deletion in the C terminal portion of nsp1 (MHV-nsp1Δ99) replicated with similar kinetics and to a similar titer as wild type virus in 17Cl-1 mouse fibroblasts. However, the nsp1 mutant was attenuated in its ability to replicate in the liver and the spleen and to cause hepatitis following IP inoculation [55]. The attenuation was not observed when IFNAR-/- mice were infected with MHV-nsp1Δ99 suggesting that nsp1 confers the ability to resist the effects of type I IFN. In support of this, there was a small but statistically significant reduction in the replication of MHV-nsp1 Δ99 in macrophages that had been pretreated with IFN-α as compared to wild type virus; however, there were no differences in resistance to IFN in cDCs and no effect on the ability of virus either to replicate or to induce IFN in macrophages or cDCs [55]. Interestingly, overexpression of SARS-CoV nsp1 (p20) inhibited RNA expression from an IFN-β promoter but a mutant nsp1 (with a deletion of the C terminal 13 amino acids) lacked this activity. Furthermore, the infection of 293T expressing the SARS-CoV receptor, ACE-2, with the deletion mutant induces IFN-β □while wild type virus does not [75]. Finally Wathelet *et al*. [76] demonstrated that over expressed SARS-CoV nsp1 could block both IFN-β induction and signaling and that an nsp1 mutant (with two amino acid substitutions) was slightly more sensitive to IFN and replicated slightly less well in Calu-3 cells as compared to wild type virus. These observations support the notion that nsp1 has an IFN antagonist activity. However, this may not be a direct effect on IFN signaling. Expression of nsp1 (or infection with wild type virus) promotes the degradation of host cell mRNA while nsp1 mutant virus does not [75]. Furthermore, overexpression of both MHV and SARS nsp1 was able to inhibit expression from reporter plasmids driven by constitutive SV40 promoters as well as the IFN-β promoter [55,75]. Thus, it is difficult to determine whether the effect on IFN-β is a direct effect of nsp1 or indirect through its ability to degrade host cell mRNA but it is clear that the nsp1 proteins of MHV and SARS-CoV are both virulence factors [30].

### Nsp3

Nsp3 is a large (180–200 kDa) multifunctional protein encoded within ORF 1a of the replicase gene of coronaviruses (Figure 1). Nsp3 contains two domains demonstrated to be virulence factors, the so called “X” or macro domain (ADP-ribose 1″-phosphatase (ADRP)) and the papain like protease (PLP) domain. PLP (or PLpro), shown to interfere with the induction of type I IFN will be discussed here and the X domain will be discussed below (Figure 1). The nsp3 of SARS-CoV was shown to be an IFN antagonist as measured by inhibition of expression of an NFkB dependent reporter plasmid [76]. The coronavirus nsp3 protein may contain one or two PLP domains. The MHV nsp3 encodes two such domains with the more C terminal one, PLP 2, functionally similar to the one PLP encoded in SARS-CoV. PLP carries out three cleavages producing nsp1–4, from the N terminal portion of the replicase polyprotein. More recently, the PLP of SARS was shown to contain a domain similar to those of cellular enzymes that have both deubiquitinating and deISGylating activities *in vitro*. It was suggested that these activities could confer a role as an IFN antagonist [77]. Several studies have demonstrated that PLP blocks IRF3 dependent induction of IFN-β; however there is disagreement about the mechanism by which this occurs. While Devaraj *et al*. [78] concluded that PLP interacts physically with IRF3 to inhibit phosphorylation, dimerization and nuclear import, Frieman *et al*. [79] confirmed that PLP inhibits the phosphorylation of IRF3 but did not detect a physical interaction between the two proteins; they demonstrated that PLP also inhibited NFκB activation by stabilizing levels of the NFκB inhibitor, IκBα. Frieman *et al*. [79] also demonstrated that while the PLP of MHV also had a deubiquitinating activity, overexpressed MHV PLP did not have the ability to interfere with IRF3 and NFκB pathways. This is in disagreement with Zheng *et al*. [80] who concluded that MHV PLP does have an IFN antagonist activity, which is dependent on the deubiquitinating activity. Finally Frieman *et al*. [79] demonstrated that the ubiquitin-like domain (UBL) present in PLP is necessary for IFN antagonism but not for protease activity and that protease activity and the deubiquitinating activities may both play roles in IFN antagonism. The relationship between the deubiquitinating activity and IFN antagonism is still not clear. However, it is quite clear that PLPs may differ among coronavirus strains in the ability to antagonize IFN induction.

### MHV virulence genes that may play roles other than compromising IFN induction or signaling

The data presented in this review provide substantial evidence that virus-host interactions leading to activation of the IFN antiviral pathway vary greatly among closely related viruses and are dependent on cell specific factors. A mutation that subtlety alters viral fitness in a particular cell type may be enough for the cellular IFN system to gain the advantage. Thus, careful consideration must be taken when proposing a direct IFN antagonizing capacity of a viral protein or ORF. With this in mind, virally encoded proteins that modulate the mode, kinetics or spatiotemporal expression of viral molecules could influence the availability of viral PAMPs for detection by PRRs or influence the sensitivity of the virus to the antiviral state induced by IFN-α/β. Thus, we include a discussion of MHV proteins or protein domains that when altered attenuate MHV pathogenesis.

### The nsp3 macro domain and ns2 putative cyclophosphodiesterase (CPD)

All coronaviruses, as well as viruses from many other groups of RNA viruses, encode a conserved ADRP domain (also referred to as the X or macro domain as above). Macro domains are ancient and conserved throughout all eukaryotic organisms including bacteria and archae suggesting they have a basic and important function. The most well characterized macro domain, histone associated MacroH2A, plays a role in cell type specific regulation of transcription [81]. In addition, MHV ns2 protein encoded in ORF2a, just downstream of the replicase gene (Figure 1), contains a domain with high homology to a superfamily of proteins known as 2H phosphoesterases and is predicted to have a 1″, 2″- cyclophosphodiesterase (CPD) activity. Mutations in each of these domains have attenuating effects on MHV *in vivo* while having no effect on replication in tissue culture. This is intriguing in that the CPD and ADRP could potentially participate in a pathway of nucleotide processing which could be involved in host interaction (as described below) [82,83]. The CPD would convert ADP-ribose-1”2” cyclic phosphate into ADP-ribose 1” phosphate. This activity has not been detected to date for the MHV ns2 protein. The ADRP of several coronaviruses including SARS, HCo-229E and porcine transmissible gastroenteritis virus was shown to convert the product of the CPD, ADP-ribose 1” phosphate, into ADP-ribose and inorganic phosphate [84,85]. In addition to its weak enzymatic properties, the ADRP also has binding activity to mono and poly ADP-ribose that potentially implicates the activity in ribosylation of host cell proteins, a posttranslational modification involved in inducing apoptosis or necrosis in response to stress [86].

Mutation of the predicted catalytic histidine residues within the putative CPD of ns2 conferred attenuation of MHV replication in the liver but not in the brain and also reduced the level of hepatitis [87]. Earlier analysis of naturally occurring ORF2a deletion within the JHM genome did not have a phenotype in mice (personal communication, Dr. Stanley Perlman, Dr. Julian Leibowitz); this is likely because JHM induces only minimal hepatitis. Interestingly, mutations of the catalytic residues of the MHV-A59 ADRP also caused attenuation of hepatitis; however this mutant virus replicated similarly to wild type in the liver and the effect on the CNS was not addressed in this study [88].

The data currently available do not support the possibility that MHV ns2 protein is able to interfere with the production or signaling of IFN-β, following virus infection. The ns2 mutants, like wild type A59 are unable to induce IFN-β, in L2 murine fibroblasts and their replication is not inhibited by IFN-β pre-treatment. Also, the mutant induces IFN-β to a similar extent as wild type following infection of bone marrow derived macrophages. It is however possible those MHV-A59 ns2 mutants are more sensitive to IFN in the relevant primary cell types *in vivo*. Similarly, mutation of the macro domain of MHV did not confer increased sensitivity to IFN-α [88]; however, this mutant did have a decreased ability to induce several cytokines, most notably IL-6, in a cell type specific manner.

The putative CPD domain of MHV is not widely conserved among coronaviruses, indicating that the hypothetical CPD-ADRP enzymatic pathway is not a common feature of this group of viruses, but may have a function for group IIA coronaviruses. The ns2 protein may have alternate or additional nonenzymatic functions; for example the phosphoesterase AKAP18, a protein kinase A anchoring protein, plays a role as a structural proteins in organization of complexes involved in signaling events [89]. In contrast to ns2, the macro domain is highly conserved among other RNA virus groups in addition to coronaviruses, implying an important function for this domain. However a recent report suggests that the macro domain of group I coronaviruses may differ from that of group III viruses in the ability to bind ADP-ribose and infer that this may imply loss of function for group III viruses or different functions for these macro domains [90]. Interestingly the Sindbis virus macro domain, a part of nsp3, has a very weak phosphatase activity. Mutation of the poly ADP ribose binding site effects neurovirulence but does not affect the binding of poly ADP-ribose. In addition poly ADP-ribose polymerase (PARP-1) binds to Sindbis nsp3 outside of the macro domain. This interaction is believed to regulate transcription in neuronal cells [91,92]. The role of the Sindbis macro domain in CNS infection, which is more likely related to its role in poly ADP-ribose binding rather than its phosphatase activity, is not yet understood.

### Nsp14

The coronavirus replicase protein nsp14 (p59), is a 3′–5′ exonuclease (ExoN) of the DEDD superfamily [83]. Mutagenesis studies have shown that the ExoN activity is essential for virus replication in cell culture. Furthermore, this activity markedly increases the fidelity of transcription of MHV conferring an unusually low mutation rate for the coronavirus replicase [93]. A single amino acid substitution Tyr6398His, 140 amino acids downstream of the last predicted exoribonuclease catalytic motif resulted in significant attenuation in mice [94]. Replication in the brain was reduced by approximately 100 fold and inoculation with as much as 10^5^ PFU failed to kill the mice. The precise role of this protein in replication and pathogenesis is not known and there are no data suggesting it interacts with the type I IFN response.

## 3. Conclusions and future questions

The type I interferon response is clearly crucial for defense against MHV infection of the two major target organs, the central nervous system and the liver, as evidenced by uncontrolled replication and accelerated mortality in MHV infected mice lacking the IFNAR. Both pDCs and macrophage/microglia are important cells for the production of type I IFN following infection. Infection of other cells types such as fibroblasts and primary neural cell types does not lead to the expression of type I IFN. Similarly the ability to resist the effects of IFN signaling is cell type specific with MHV being relatively resistant to IFN in fibroblast cell lines, while exhibiting sensitivity in macrophages and MEFs. Indeed, it is IFN signaling in macrophages that is most important for protection against MHV infection by the IP route of inoculation [47]. The mechanisms behind the cell type specific differences in IFN induction and signaling are not well understood and may involve differences in expression of PRRs, transcription factors, and other mediators as well as different routes of infection. This idea is supported by evidence that all cell types investigated are capable of inhibiting replication of other RNA viruses in response to type I IFN. Resistance of MHV to the antiviral effects of IFN may be at least in part due to IFN antagonists; as described above, there are studies suggesting that nsp1, nsp3 and N have antagonistic roles in IFN induction and signaling. However, the mechanisms by which IFN antagonism occurs during MHV infection is not well understood and merits further investigation.

Future studies will be directed toward understanding the cell type specific differences in IFN induction and signaling. It will be important to understand the role of IFN in protection from MHV infection in the CNS and the liver, the major target organs of MHV. More specifically, we plan investigate how non-renewable cells such as neurons which do not produce IFN upon infection, are protected against virus, perhaps by basal high levels of ISGs. We will also investigate a role for IFN in the inability of JHM to infect the liver despite the fact that its receptor is more highly expressed in the liver than the brain and it can infect hepatocytes *in vitro*.
